# The drivers of antibiotic use and misuse: the development and investigation of a theory driven community measure

**DOI:** 10.1186/s12889-019-7796-8

**Published:** 2019-10-30

**Authors:** Mitchell K. Byrne, Sebastien Miellet, Anica McGlinn, Janaye Fish, Shahla Meedya, Nina Reynolds, Antoine M. van Oijen

**Affiliations:** 1 School of Psychology, University of Wollongong, Building 41, Northfields Ave, Wollongong, NSW 2522 Australia; 2 Wollongong Antimicrobial Resistance Research Alliance (WARRA), Wollongong, New South Wales Australia; 3 Research Department, Illawarra Shoalhaven Local Health District, New South Wales, Australia; 4 School of Nursing, University of Wollongong, Wollongong, New South Wales Australia; 5 Molecular Horizons Institute and School of Chemistry and Biomolecular Science, University of Wollongong, Wollongong, New South Wales Australia; 6 Illawarra Health and Medical Research Institute, Wollongong, New South Wales Australia; 7 School of Management, Operations and Marketing, University of Wollongong, Wollongong, New South Wales Australia

**Keywords:** Antibiotic, Antibiotic use, Antimicrobial resistance, Attitude, Behaviour change, Social theory, Public health, Measurement, Psychometrics

## Abstract

**Background:**

Antimicrobial resistance is a global public health concern, with extensive associated health and economic implications. Actions to slow and contain the development of resistance are imperative. Despite the fact that overuse and misuse of antibiotics are highlighted as major contributing factors to this resistance, no sufficiently validated measures aiming to investigate the drivers behind consumer behaviour amongst the general population are available. The objective of this study was to develop and investigate the psychometric properties of an original, novel and multiple-item questionnaire, informed by the Theory of Planned Behaviour, to measure factors contributing to self-reported antibiotic use within the community.

**Method:**

A three-phase process was employed, including literature review and item generation; expert panel review; and pre-test. Investigation of the questionnaire was subsequently conducted through a cross-sectional, anonymous survey. Orthogonal principal analysis with varimax rotation, cronbach alpha and linear mixed-effects modelling analyses were conducted. A 60 item questionnaire was produced encompassing demographics, social desirability, three constructs of the Theory of Planned Behaviour including: attitudes and beliefs; subjective norm; perceived behavioural control; behaviour; and a covariate – knowledge.

**Results:**

Three hundred seventy-three participants completed the survey. Eighty participants (21%) were excluded due to social desirability concerns, with data from the remaining 293 participants analysed. Results showed modest but acceptable levels of internal reliability, with high inter-item correlations within each construct. All four variables and the outcome variable of antibiotic use behaviour comprised four items with the exception of social norms, for which there were two items, producing a final 18 item questionnaire. Perceived behavioural control, social norms, the interaction between attitudes and beliefs and knowledge, and the presence of a healthcare worker in the family were all significant predictors of antibiotic use behaviour. All other predictors tested produced a nonsignificant relationship with the outcome variable of self-reported antibiotic use.

**Conclusion:**

This study successfully developed and validated a novel tool which assesses factors influencing community antibiotic use and misuse. The questionnaire can be used to guide appropriate intervention strategies to reduce antibiotic misuse in the general population. Future research is required to assess the extent to which this tool can guide community-based intervention strategies.

## Introduction

Antibiotics are an antimicrobial agent defined as “a chemical substance produced by a microorganism that kills or inhibits the growth of another microorganism” [1]. Since the introduction of the first effective antimicrobial in 1937 [2], there has been persistent growth and spread of drug-resistant bacteria, broadly referred to as antimicrobial resistance (AMR). AMR is defined as the phenomenon where infection-causing microorganisms, such as bacteria, have the ability to survive exposure to medicine which would normally inhibit their growth or kill them [3]. The health implications of AMR are extensive, affecting not only the treatment of a primary bacterial infection, but also the prophylactic use of antibiotics in routine surgical procedures, such as caesareans and hip replacements [3, 4]. O’Neill (2016) estimates that, unchecked, the growth of AMR will result in 10 million preventable deaths per year by 2050. In addition to the human cost, the increase in AMR is associated with significant economic consequences [5]. AMR is associated with increased expenditure on health services, with greater resource utilisation and higher levels of routine health care costs [6–8]. The additional impact of AMR has downstream effects on health service productivity [9]. Unfettered, it is estimated that by 2050, AMR will have impacted world global production by $US100 trillion [3].

From an evolutionary standpoint, AMR is unavoidable [10] due to bacteria’s inherent ability to survive, mutate and adapt, following stress and greater exposure to antimicrobials [4]. Given that AMR cannot be reversed or eradicated [11], actions to slow and contain the development of resistance are imperative [12]. The rate of AMR development is widely understood to be facilitated by indiscriminate and unnecessary antibiotic use [3, 13–16]. The World Health Organisation (WHO) Global Strategy for Containment of Antimicrobial Resistance (2001) defines *appropriate* antimicrobial use as the “cost effective use of antimicrobials which maximises clinical therapeutic effect whilst minimising drug-related toxicity and development of antimicrobial resistance” [17].

Existing literature highlights consumer or patient demand and behaviour, as a driving force behind antibiotic misuse [18–20]. Understanding the extent of global trends in consumer demand for, and knowledge about, antibiotics is therefore an important component in the battle to curtail the growth of AMR and has precipitated multinational surveys. For example, a survey carried out by the Taylor Nelson Sofres (TNS) Opinion and Social for the European Commission (2010) gathered information from 26,761 individuals across the (then) 27 member states of the European Union. The survey found that 40% of respondents had taken antibiotics in the previous 12 months, with 95% reporting that they (appropriately) obtained them from a medical practitioner. However, the survey also reported that only 20% of respondents were able to correctly answer four knowledge statements regarding antibiotics, including 53% who believed that antibiotics kill viruses, and 47% who believed antibiotics were effective against colds and influenza. These results suggest that while Europeans report obtaining antibiotics through appropriate means (doctors), their intended use is often inappropriate [21].

A subsequent survey conducted by the WHO (2015) questioned 9772 individuals across two member states in the six WHO regions. In this survey it was found that 65% of respondents had used antibiotics in the previous 6 months, with 81% (range 56–93%) indicating that they had obtained them from a medical professional. The WHO survey reported that 25% of respondents believed it acceptable to use antibiotics given to them by a friend or family member, 43% thought it acceptable to buy antibiotics or seek them from a doctor if they were sick with symptoms that they believed were effectively treated by antibiotics in the past, and 64% incorrectly believed viruses such as colds and influenza could be treated by antibiotics [15].

According to Wise et al., (1998), 20% of human antibiotic use occurs within the hospital sector, whilst 80% is within the community sector. Within this community portion, 20–50% may be questionable and unnecessary [22]. Within Australia specifically, antibiotic consumption rate exceeds the Organisation for Economic Cooperation and Development (OECD) average [23]. Thus, an understanding of the drivers of Australian consumer antibiotic seeking and use is warranted.

Both the TNS Opinion & Social (2010) and WHO (2015) surveys had numerous limitations, including various sampling techniques, bias toward more educated responders, and an absence of checks upon socially desirable responding. Furthermore, neither survey was theory informed in order to enable prediction of consumer antibiotic use, other than the potential impact of poor knowledge about antibiotics and AMR, and neither reported detailed psychometric properties of the questionnaires. They do, however, confirm previous research which has identified a range of key factors contributing to patient behaviour with respect to antibiotic use, including attitudes and beliefs, subjective norms, self-efficacy and knowledge [6, 24].

There are few measures which currently exist in this area. Many are specific to population sub-groups, including physicians, parents [6, 25–27], medical students [28] and pharmacists [29, 30]. To our knowledge, there exists no sufficiently validated measure which aims to investigate factors influencing antibiotic use within the general populace [31].

**Fig. 1:**
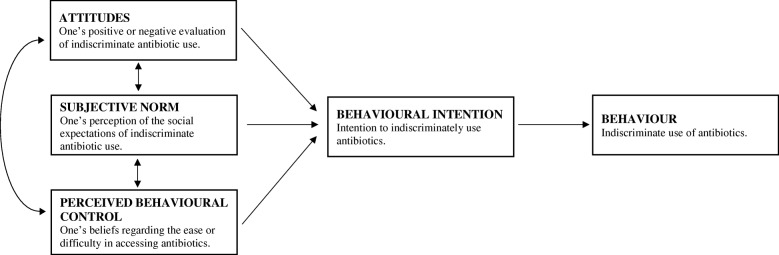
Theory of Planned Behaviour model, adapted from Ajzen (1986)

The current study sought to develop a questionnaire that predicts the factors influencing a consumer’s intentions to indiscriminately obtain and use antibiotics. Given that attitudes and beliefs [32], the opinions of others within a person’s social or professional network [33–35], and the self-perceived (and actual) ability to obtain antibiotics [36–38] have all been independently associated with the use of antibiotics, the current study aimed to construct a questionnaire informed by the Theory of Planned Behaviour (TpB) [39], a respected and highly cited model which predicts health related behaviours [40–43]. The TpB, which has yet to be used in the context of antibiotic use for consumers, would suggest that a person’s actual use of antibiotics is best predicted by their intentions, which are influenced by three major components (see Fig. 1): (a) attitudes, referring to one’s positive or negative evaluation of indiscriminate antibiotic use, (e.g. ‘the negatives of taking antibiotics outweigh the positives’); (b) subjective norm, involving their perception of the social expectations of indiscriminate antibiotic use, (e.g. ‘my friends and family would follow recommendations for antibiotic use’); and (c) perceived behavioural control (PBC), reflecting the beliefs regarding the ease or difficulty in accessing antibiotics, (e.g. ‘I would easily be able to get antibiotics if I wanted them’). PBC was the only control measure as actual behavioural control (added to later TpB models) [44] was unable to be measured within this study protocol. A condition of the strength of this PBC-behaviour relationship is that ‘perceptions of behavioural control must reflect actual control in the situation with some degree of accuracy’. When perceptions of control are accurate, PBC is expected to predict behaviour [45–47].

One of the most extensive TpB reviews, focusing on prospective behaviours across 237 studies, was conducted by McEachan, Conner, Taylor and Lawton, [48], who found that the TpB could explain 19.3% of variance in behaviour and 44.3% of the variance in intention to behave. McEachan and colleagues further demonstrated that the TpB provides strong predictions of intention and behaviour across a range of health behaviours, with the attitude component being the strongest behavioural intention predictor. Further, Ajzen, (1991) suggests that the TpB is highly adaptive, possessing the ability to incorporate additional predictors where required, providing that they maintain the ability to capture a significant proportion of variance in intention or behaviour, and also given that the initial variables have been considered. Given previous research, knowledge about antimicrobials, and AMR specifically, would be expected to influence attitudes [32].

Limitations surrounding the TpB include its sole reliance upon self-reported behaviour, potentially inspiring socially desirable and less accurate predictions of objective behaviour [47]. Armitage and Conner undertook a meta-analysis of 161 articles containing 185 independent empirical tests of the TpB, concluding that the use of the model is effective in predicting intention and behaviour, more so in the context of subjective self-reported behaviour over observed behaviour (R-squared 0.31 and 0.20 respective) [40]. This is not a limitation specific to the TpB, but broadly to the area of social psychology, and is not a large cause for concern given the model still capably measures a good amount of variance in prospective measures of actual behaviour [40]. Moreover, the TpB showcases high consistency between intention and behaviour, even in contexts of differing emotional states [47]. None-the-less, attention to social desirability would enhance the predictive validity of the TpB as applied to consumer antibiotic use.

Thus, the aim of the current study is to develop and investigate the psychometric properties of an original, novel and multiple-item quantitative questionnaire, aiming to identify factors contributing to antibiotic use within the community, informed by the TpB. Considering the adaptive nature of the TpB [39], knowledge was added as a variable of interest within the current study, due to the array of literature which indicates a relationship between knowledge and antibiotic-use behaviour [31].

## Methods

### Questionnaire development

A three-phase process was employed to develop the Antibiotic Use Questionnaire (AUQ) utilised within this study. Phases included: a literature review and item generation; expert panel review; and pre-test. Investigation of the AUQ was then subsequently conducted through a cross-sectional, anonymous and voluntary survey.

### Phase one. Literature review and item generation

An opening list of 43 items were drawn from a literature review of previous studies investigating consumer characteristics and self-reported antibiotic use, using search terms such as ‘antibiotic use’, ‘AMR’, and ‘antibiotic use influences’. Questions were then grouped under discrete categories, including demographics, knowledge, TpB constructs (attitude, subjective norm and PBC), and an outcome factor - self-reported antibiotic use behaviour. Items were adapted where required to suit the cultural and sociodemographic context of the target population. For example, ‘Aboriginal or Torres Strait Islander’ was added as an option for ancestry to reflect the Australian population.

### Phase two. Expert panel review

The original 43 item questionnaire was examined for content validity by a panel of eight experts, organised to represent a range of fields including psychology, business and health. Questions were evaluated with respect to the extent to which, on face value, they aligned with the TpB variables (including knowledge), their repetition, clarity and cultural relevance. Additional questions were generated in areas under-represented, such as social norms, and redundant questions removed. A subset of six items from the Marlowe-Crowne Social Desirability Scale [49] were randomly selected and incorporated into the questionnaire, to allow for measurement of the honesty and reliability in respondent answers. Following agreement by the expert panel on the established questions, selected items (excluding demographics) were randomised using Stat Trek: Random Number Generator (no date) to mediate response bias. Review of the questionnaire involved 10 iterations with the expert panel and yielded an initial (pre-assessment) questionnaire of 60 items, organised as per Table 1, with questions requiring multiple choice, dichotomous or likert scaled responses.

**Table 1:** Organisation of the initial (pre-assessment) AUQ

Variable	Description	Instrument Items	Example
Demographics	Data relating to the characteristics of a participant.	12 items	‘Are you trained in a health-related field?’ ‘How often have you used antibiotics within the past month?’
Social Desirability	A participant’s tendency to respond in a way they deem more socially acceptable, than their ‘true’ response.	6 items	‘I have never deliberately said something that hurt someone’s feelings’ ‘There have been times when I was quite jealous of the good fortune of others’
TpB Construct			
Attitude and Beliefs	The degree to which a participant has a positive or negative evaluation of indiscriminate antibiotic use.	13 items	‘It is my right to ask for an antibiotic from my doctor’ ‘I trust my doctor when they tell me I do not require antibiotics’
Subjective Norm	A participant’s belief about whether significant others would approve or disapprove of indiscriminate antibiotic use.	4 items	‘My friends and family only use antibiotics when prescribed’ ‘Most people I know keep leftover antibiotics’
Perceived Behavioural Control	A participants beliefs regarding the ease or difficulty in assessing antibiotics.	5 items	‘I would change doctors if my doctor did not prescribe antibiotics when I wanted them’ ‘I feel confident to ask for antibiotics when I need them’ ‘I could easily get antibiotics online’
Knowledge*	A participants understanding and awareness regarding indiscriminate antibiotic use and AMR.	10 items	‘Antibiotics will reduce my cold symptoms’ ‘Antibiotics are less likely to work in the future’ ‘The same antibiotic will work in the treatment of the same infection in the future’
Behaviour	Self-reported antibiotic use behaviour.	10 items	‘I obtain antibiotics without a prescription’ ‘I would take antibiotics without consulting a doctor’ ‘I use leftover or unused antibiotics or scripts’

*Added as a construct to the TpB model for the purposes of this study

### Phase three. Pre-test

Before administration of the questionnaire to participants, a group of 10 participants pre-tested the survey to examine face validity. Feedback was gathered on time to completion, question clarity, perceived relevance, and face validity. Minor adjustments were made based on feedback received.

### Data collection and ethics

The finalised questionnaire was distributed via an anonymous cross-sectional survey conducted between July – August 2018. Tacit consent was obtained, inferred through anonymous completion and return of the questionnaire. Survey Monkey was used to create a soft copy version of the questionnaire, with the link being distributed via non-moderated e-mail services and social media, predominantly incorporating snowballing techniques. Hard copy questionnaires were also distributed, mainly to participants who were unable to be reached via e-mail or social media, and for the purposes of purposive sampling after a mid-data collection review identified disparities in demographic representation. All hard copy questionnaires were completed in the presence of a researcher. Purposive sampling took place in popular public spaces, including a local shopping mall and retirement club, with a desire to achieve balance from older age groups, males, and those of lower socioeconomic status and education level. Hardcopies were returned directly to the researcher after completion, and manually entered into a Microsoft Office Excel, Version 10, spread sheet. The current study was approved by the Health and Medical Human Research Ethics Committee (Joint University of Wollongong and Illawarra Shoalhaven Local Health District, 2018/330).

### Sample

All recipients of the questionnaire, aged 18 years and over, were invited to partake in the research. Completed questionnaires were received from 373 participants. The majority of participants were recruited via online platforms (91%, *n* = 338), with the remaining participants recruited in person (9%, *n* = 34). Eighty participants (21%) were excluded from the analysis, due to concerns regarding the accuracy and reliability of their responses, after scoring equal to or higher than five in social desirability. Data from the remaining 293 (79%) participants was analysed.

### Data analysis

Data analysis was carried out using Matlab R2018A (The MathWorks Inc). All completed questionnaires were screened for missing data, outliers and coding errors. Participants answered at least 83% of the questions (mean = 99.24%, standard deviation (SD) =3.23). Descriptive statistics for patient demographics were reported, expressed as raw numbers and percentages. An orthogonal principal component analysis with varimax rotation (factoran Matlab function) was utilised to assess the factor loadings of the questionnaire items for the four dimensions of the TpB and the covariate – knowledge. Cronbach alpha was used to determine the internal reliability of items relating to each of the five factors. Furthermore, linear mixed-effects modelling was employed to study the influence of the TpB factors on intended antibiotic use behaviour. The moderation from knowledge on the link between attitude/belief and intended behaviour was modelled as an interaction term between knowledge and attitude/belief. The fixed effects of the model included the interaction between attitudes and beliefs, and knowledge, PBC, social norms, age, gender, education, whether the participants had children, health trained, health worker in the family, frequency of antibiotic consumption, financial security, and most recent antibiotic consumption. The random effect was the participants.

## Results

Participant demographics are reported in Table 2. Mean score for social desirability (range 0 to 6) was 2.69 (±1.16). Of the 80 (21%) participants who were excluded due to a high social desirability score (5 or above), 65% (*n* = 52) were female with 55% (*n* = 44) of respondents having a bachelor degree qualification or higher. 59% (*n* = 47) of respondents were aged between 18 and 44 years, with the 18–24 year category the highest (*n* = 25). Whilst majority of respondents were not personally trained in a health-related field (68%, *n* = 54), 58% (*n* = 46) had a family member or friend with a health-related occupation. 55% (n = 44) of respondents excluded had not taken an antibiotic within the past year.

**Table 2:** Participant characteristics

Characteristic	n*	%
Age		
18–24	124	42.5%
25–34	49	16.8%
35–44	28	9.6%
45–54	32	11.0%
55–64	34	11.6%
65–74	9	3.1%
75+	16	5.5%
Gender		
Male	106	36.2%
Female	186	63.5%
Other	1	0.3%
Ancestry^#^		
Australian	190	64.8%
Aboriginal or Torres Strait Islander	8	2.7%
Chinese	11	3.8%
English	77	26.3%
German	17	5.8%
Irish	29	9.9%
Italian	9	3.1%
Scottish	26	8.9%
Other	66	22.5%
Education level		
Secondary School, did not complete year 12	17	5.8%
Secondary School, completed year 12	59	20.1%
TAFE	47	16.0%
Bachelor’s Degree	125	42.7%
Postgraduate Degree, i.e. Masters or PhD	45	15.4%
Whether Participants had Children		
Yes	104	35.5%
No	189	64.5%
Financial security		
Strongly Disagree	13	4.4%
Disagree	45	15.4%
Agree	180	61.4%
Strongly Agree	55	18.8%
Personal training in a health-related field		
Yes	87	29.7%
No	206	70.3%
Family member or friend with a health-related occupation		
Yes	178	61.0%
No	114	39.0%

*Number may not add up to 293 due to missing values. ^#^Multiple responses allowed

Of the remaining 293 participants whose data was included, majority (74%, *n* = 217) identified themselves as infrequent antibiotic users, consuming antibiotics once a year or less, with less than a third of respondents (30%, *n* = 87) consuming antibiotics within the past 6 months. Consistent with previous research, although 83% (*n* = 242) of participants correctly identified that antibiotics should be used for the treatment of bacterial infections, 25% (*n* = 74) of these respondents also incorrectly identified that they work for viral infections and/or fungal infections.

Factor loadings of the questionnaire items for the three variables of the TpB, the outcome variable (behaviour), and covariate, knowledge, are reported in Table 3. All five variables encompassed four items with the exception of social norms, which included two items, yielding a final 18-item questionnaire.

**Table 3:** Factor loadings for questionnaire items*

Item Number and Item	Attitude and Beliefs	Social Norms	PBC	Knowledge	Behaviour
Item 1. Antibiotics will reduce my cold symptoms	0.138	0.093	0.136	**0.769**	0.044
Item 2. My friends and family follow recommendations for antibiotic use	−0.036	**0.552**	−0.052	0.121	0.016
Item 3. Antibiotics are needed for the common cold	0.081	0.029	−0.001	**0.695**	0.050
Item 4. Antibiotics may have negative side effects	0.137	0.282	−0.089	**− 0.294**	− 0.013
Item 5. I would take antibiotics without consulting a doctor	0.295	−0.191	0.195	0.090	**0.561**
Item 6. I use leftover or unused antibiotics or scripts	0.149	0.032	0.110	0.090	**0.830**
Item 7. It is my right to ask for an antibiotic from my doctor	**0.423**	0.079	−0.019	0.158	0.071
Item 8. My friends and family only use antibiotics when prescribed	−0.010	**0.698**	−0.185	0.006	−0.151
Item 9. I know I need antibiotics before I see my doctor	**0.693**	0.003	0.000	−0.082	0.231
Item 10. In my community, it is common to use antibiotics without a prescription	−0.044	−0.255	**0.390**	0.075	0.119
Item 11. I feel confident to ask for antibiotics when I need them	**0.505**	0.004	0.155	−0.054	0.075
Item 12. Antibiotics are less likely to work in the future	0.011	−0.002	0.027	**−0.410**	−0.093
Item 13. I consult with my doctor prior to taking antibiotics	−0.082	0.338	−0.009	−0.217	**− 0.544**
Item 14. I keep leftover or unused antibiotics or scripts	0.187	0.040	0.078	0.008	**0.602**
Item 15. I could easily get antibiotics from a doctor	0.303	−0.043	**0.358**	−0.018	0.029
Item 16. I could easily get antibiotics online	0.071	−0.038	**0.517**	0.037	0.011
Item 17. I could easily get antibiotics from my family / a friend / household	0.037	−0.106	**0.884**	0.010	0.191
Item 18. By the time I am sick enough to see my doctor, I expect a prescription of antibiotics	**0.408**	−0.108	0.037	0.131	0.226

*Bold font indicates which factor the items are associated with

Results showed modest but acceptable levels of internal reliability (Cronbach alpha) within each variable: attitudes and beliefs = 0.60; social norms = 0.59; PBC = 0.64; knowledge = 0.61; behaviour = 0.76 [50]. Inter-item correlations where high within each variable (Attitudes and beliefs = 0.28; social norms = 0.42; PBC = 0.30; knowledge = 0.28; behaviour = 0.45). In contrast, inter-item correlations were low between variables (0.11 on average).

A Linear-mixed model was run with the following fixed effects: the interaction between attitudes and beliefs and knowledge; PBC; social norms; age; gender; education; whether the participants had children, were health trained, had a health worker in the family; frequency of antibiotic consumption; financial security; and most recent antibiotic consumption. The random effect was the participants. Fixed effects coefficients can be found in Table 4. For this model the Akaike Information Criterion (AIC) was 1033.5 and the Bayesian Information Criterion (BIC) was 1088.7. The ordinary R-squared was 0.7071 and the adjusted R-squared was 0.6945; indicating that this model explains around 70% of the variance in the self-reported antibiotic misuse. The part of variance explained by the model decreases when knowledge is not included (ordinary R-squared: 0.6950; adjusted R-squared: 0.6820). The modest decrease could be explained by the other fixed effects capturing partially the part of variance that was explained by knowledge; thus indicating some relative overlap between these constructs as illustrated by the loading factors in the factor analysis. The fixed effect variables, PBC (β = −.22, *p* = 0.001), Social Norms (β = .24, *p* = 0.047), interaction between attitudes and beliefs and knowledge (β = .09, *p* = < 0.001), and the presence of a healthcare worker in the family (β = .35, *p* = 0.039), were all significant predictors of antibiotic use behaviour. All other predictors tested did not produce a significant relationship with the outcome variable.

**Table 4:** Fixed effects coefficients

Name	Estimate	Standard Error	t-Statistic	Degrees of Freedom	*p*-Value	Lower	Upper
Intercept	2.404	1.090	2.206	280	0.028	0.259	4.548
Interaction between attitudes, beliefs and knowledge	0.088	0.013	6.813	280	5.834e-11	0.062	0.113
Perceived behavioural control	−0.220	0.067	−3.298	280	0.001	−0.351	−0.089
Social norms	0.243	0.122	1.991	280	0.048	0.003	0.483
Age	−0.012	0.067	−0.176	280	0.861	−0.143	0.119
Gender	−0.177	0.177	−1.005	280	0.316	−0.525	0.170
Education level	0.020	0.073	0.269	280	0.788	−0.124	0.163
Whether participants had children	0.126	0.234	0.539	280	0.590	−0.334	0.586
Personal training in a health-related field	−0.126	0.184	−0.688	280	0.492	−0.488	0.235
Family member or friend with a health-related occupation	0.346	0.167	2.079	280	0.039	0.018	0.674
Frequency of antibiotic use	0.132	0.092	1.435	280	0.152	−0.049	0.313
Perception of financial security	−0.011	0.120	−0.092	280	0.927	−0.247	0.225
Last occasion of antibiotic use	0.074	0.086	0.855	280	0.393	−0.096	0.244

An alternative model was tested with the same variables except for the moderator knowledge. AIC and BIC were slightly higher in this case (1040.9 and 1096.1 respectively) and the model explained a smaller proportion of variance (ordinary and adjusted R-squared: 0.6950 and 0.6820).

**Fig. 2:**
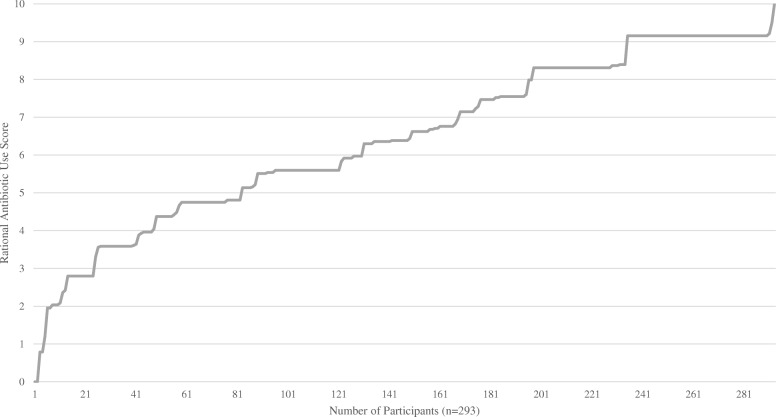
Scores of rational antibiotic use for all participants (sorted)

Scores for rational antibiotic use were calculated using the factor loading coefficients from the questionnaire items which loaded to the construct of behaviour. Calculations were made as follows: 0.561*Behaviour Item 5 + 0.83*Behaviour Item 6–0.544*Behaviour Item 13 + 0.602*Behaviour Item 14. Scores were subsequently normalised in order to have 0 as the minimal score, and 10 as the maximum score. High scores are reflective of rational antibiotic use, whilst low scores are reflective of less rational behaviour. Figure 2 outlines scores of rational antibiotic use for all participants.

## Discussion

The aim of the present study was to develop and investigate a novel quantitative measure, modelled on the TpB. The study sought to assess the factors influencing community antibiotic use and misuse, including: TpB variables (attitudes and beliefs, subjective norm and PBC); knowledge; and key demographic characteristics (such as age, gender, education level, financial status, the presence of offspring and personal health-related field training).

The confirmatory factor analysis identified items corresponding to the three variables of the TpB, the outcome variable (behaviour), and the covariate knowledge. The selected items demonstrated good psychometric properties in terms of internal reliability and convergent and discriminant validity. The internal reliability values are particularly encouraging considering the small number of items and the fact that when utilising Cronbach’s Alpha as a measure of internal consistency, the greater the number of items in the pool, the better the chance of obtaining a positive value, indicating greater internal consistency [51].

A linear-mixed effects analysis revealed that intent of antibiotic use behaviour can significantly be explained by each of the TpB variables (PBC, social norms and attitudes and beliefs moderated by knowledge) and that the TpB construct predicted 70% of the variance in antibiotic use and misuse. This amount of predicted variance exceeds that of past literature using the TpB model to predict health related behaviours [39–41, 48], and supports the use of the TpB model in this context. The presence of a healthcare worker in the family was also a significant predictor of antibiotic use behaviour. Contrary to previous research, demographic variables such level of education in this study did not significantly predict intention to seek and use antibiotics [52].

To our knowledge, this is the first sufficiently validated measure which assesses factors influencing antibiotic use and misuse within a general population and the first application of the TpB to the prediction of antibiotic use behaviour. The measure provides an opportunity for targeted intervention programs to reduce antibiotic misuse in the general community, and may inform public policy decisions. For example, while O’Neill has observed that greater accessibility to antibiotics is associated with an increase in indiscriminate use, there are limited empirical studies exploring this relationship [3]. Our finding that PBC is associated with antibiotic use behaviours confirms the observations of O’Neill [3].

Our study also contributes to an understanding of the role of knowledge, and hence the value of public educational programs, on antibiotic use intentions. Previous research into this relationship is largely contradictory; whilst some indicate a relationship between lesser antibiotic knowledge and more indiscriminate antibiotic use [13, 25], others relate greater knowledge to more indiscriminate use [53], and some do not identify a relationship between the two at all [54–56].

In our study we observed an interaction effect between knowledge and attitude/beliefs. This finding may provide evidence as to why information-intensive or educational-driven interventions alone are not entirely efficacious or maintain long-lasting results [57, 58], and likely require a multi-factorial approach, targeting the range of motivating factors which contribute to antibiotic use, i.e. attitudes and beliefs, behavioural control and knowledge. As indicated by Edgar, Boyd and Palamè, behaviour change is unlikely unless motivating factors, values and subjective norm cumulatively encourage that change [57].

Consistent with the TpB, subjective norm contributed to the prediction of antibiotic use behaviours, suggesting that antibiotic use behaviours are influenced by peers, family and community/cultural factors. This is a complex relationship given that in our study we found that the presence of family or friends working in a health-related field is associated with indiscriminate antibiotic use. Scaioli et al., indicated a similar finding, whereby those with a family member working in a health-related field were more likely to use non-prescribed antibiotics and keep left-over antibiotics [56]. It is likely that this relationship is associated with access to antibiotics (PBC) and requires further investigation.

The remaining demographic variables did not significantly influence self-reported antibiotic-use behaviour. These results are not entirely surprising, given that the current literature is contradictory when examining the relationship between demographic variables and antibiotic use. Conflicting findings in the context of antibiotic-use behaviour are apparent for education level [53, 59, 60]; income and socioeconomic status [29, 53, 61]; gender [53, 59] and age [62, 63]. Although the reason for this inconsistency is currently unclear, it may be hypothesised that the differing geographic locations, healthcare regulations and policies of the differing countries where these studies are based may be a contributing factor, although future research is needed to investigate this. The present study, while providing a novel and important lens upon which to examine the AMR dilemma, has several limitations. First, the confusing results associated with the contribution of subjective norms requires a deeper investigation. In this study the questionnaire contained only 2 items and had the weakest internal consistency. Further research is required to uncover and input additional items which aim to target subjective norm, and which enable analysis of the relationship between PCB and subjective norm, is required.

Secondly, the sample size and representation is modest. This might explain why there was not a greater relationship between demographic variables and predictors of antibiotic use behaviours. A replication of this study with a larger and more representative sample would add to the value of the AUQ. Related to this, the questionnaire was distributed to an Australian sample and may not generalise to other nations or cultures. Further research is needed to determine whether the relationships identified in this study are replicated in other international samples.

Finally, the utility of the study in informing intervention programs needs to be tested. This relates in particular to the verification of antibiotic use *intent*, and actual antibiotic use behaviours. Application of the AUQ to a population cohort and an informed intervention based on the identified drivers of antibiotic use behaviours is required.

## Conclusion

This study successfully developed and validated an original, theory driven tool which assesses factors influencing community antibiotic use and misuse. Notwithstanding the above mentioned limitations, the research highlights the pervasive influence that people-driven factors have upon antibiotic-use behaviours, likely contributing to the growth of AMR on a widespread scale. Furthermore, these findings have implications for the development of sustainable, multi-dimensional interventions that reflect the multitude of factors influencing antibiotic misuse. While AMR is a multifactorial problem requiring intervention at many levels of policy, drug discovery, and molecular biology, the role of end-point users, consumers, is a vital component of the worldwide effort to address AMR.

## Data Availability

The datasets used and/or analysed during the current study are available from the corresponding author on reasonable request.

## Abbreviations

AIC: Akaike Information Criterion
AMR: Antimicrobial resistance
AUQ: Antibiotic Use Questionnaire
BIC: Bayesian Information Criterion
OECD: Organisation for Economic Cooperation and Development
PBC: Perceived behavioural control
SD: Standard deviation
TNS: Taylor Nelson Sofres
TpB: Theory of Planned Behaviour
WARRA: Wollongong Antimicrobial Resistance Research Alliance
WHO: World Health Organisation
